# A method for rapid assessment of the distribution and conservation status of Indian pangolin (*Manis crassicaudata*) in an extended geographical region

**DOI:** 10.1016/j.mex.2020.100912

**Published:** 2020-05-15

**Authors:** Hasitha Karawita, Priyan Perera

**Affiliations:** a Department of Forestry and Environmental Science, University of Sri Jayewardenepura, Sri Lanka; b IUCN SSC Pangolin Specialist Group, C/o Zoological Society of London, Regent's Park, London NW1 4RY, United Kingdom

**Keywords:** Crime, Rescue, Distribution, Habitat, Indian pangolin

## Abstract

Development of an effective conservation management strategy for the Indian pangolins (*Manis crassicaudata*) found in Sri Lanka is hindered due to lack of solid evidence based distribution and ecological data on Indian pangolins. We employed a rapid and cost-effective method based on reliable information by combining primary and secondary data. The method was predominantly based on secondary data from the official records maintained by the government and non-governmental institutions related to wildlife conservation. The primary data collection was carried out depending on the findings from the secondary data sources; i.e. structured interviews and field studies were carried out in the localities that identified from secondary data sources. As a source of primary data, the structured interviews were carried out with stakeholders including the officials of government and nongovernmental institutions, hunters and villagers of the identified localities.

• This method allows collecting quick and accurate data on the distribution, habitats and conservation threats for the species.
• Cost effective method to collect ecological data of elusive mammals in large areas.
• Efficient method to identify trends of pangolin related crimes and illicit trade.

## Graphical abstract

**Figure:**
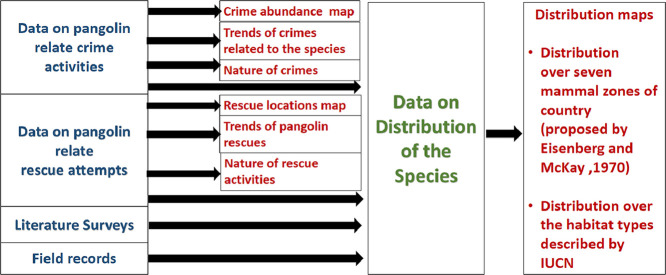

**Table:** Specifications Table

Subject area:	Environmental science
More specific subject area:	Ecology and conservation
Method name:	Assessment of distribution and conservation status
Name and reference of original method:	Adapted from several methodologies referenced in the article
Resource availability:	Perera, P., & Karawita, H. (2020). An update of distribution, habitats and conservation status of the Indian pangolin (Manis crassicaudata) in Sri Lanka. *Global Ecology and Conservation, 21*, e00799.

## Method

### Systematic review of literature and field surveys

**Fig. 1:**
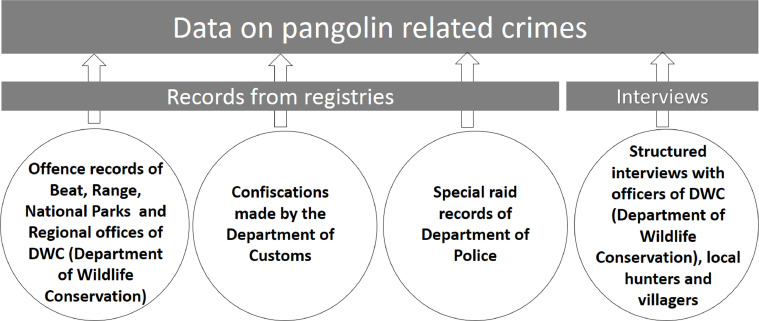
Method used to collect data on pangolin related crimes.

Lack of baseline ecological data on the Indian pangolins found in Sri Lanka is the major impediment of identifying a specific method to collect the distribution, crime and rescue data related to the pangolins. Therefore a basic literature survey was carried out at the outset of the study to develop a methodology to collect the data [1]. A preliminary literature survey was conducted on Indian pangolins found in Sri Lanka and identified the recorded localities, related crimes, rescue activities, and many other related aspects. The literature survey was carried out using the published research articles, web based articles, reliable unpublished records, field journals/records maintained by the field researchers and organizations working for wildlife conservation and the library catalogs of intuitions which maintain wildlife research records [2,3]. The web-based search was carried out using the key words “Indian pangolin” and “Pangolins of Sri Lanka” to find the articles written in English. The web articles written in local languages were searched using the keywords of “kaballǣvā” and “Eṟumpuṇṇi”; respectively, indicating the Sinhala and Tamil translations of the term ‘pangolin’. In order to find published literature, many online indexing databases such as Scopus, Web of Science and Google Scholar were searched (Fig. 1) [4,5]. Web based data from reliable sources have been widely used in evidence based conservation efforts to gain a holistic understanding on the issues related to the species to be conserved and get connected with the right group of experts [6]. The data mining from online sources using vernacular and scientific name become common in monitoring international wildlife trafficking, hence physical observations and surveys are inapplicable in regional or global scale [7]. Through the preliminary literature surveys, the registries maintained by the stakeholder institutions such as the Department of Wildlife Conservation, Department of National Zoological Gardens, Department of Customs, and Department of Police were identified as secondary data sources. In an urgent need of ecological data related to the distribution and conservation status of regionally or globally threatened species, the secondary data from the stakeholder groups or institutions are critically important [8]. Field surveys were mainly carried out in three study sites located in Yagirala (6°21′ to 6°26′ N and 80°08′ to 80°11′ E), Wilpattu (°13′ to 8°40′N and 79°50′ to 80°10′E) and Yala (6°18′ to 6°42′N and 81°24 to 81°43′E), Sri Lanka from August 2013 to December, 2018 (Fig. 1). The Field surveys include the habitat characterization, study of feeding ecology [9,10], camera trapping studies and abundance estimation [11,12] in forest associated landscapes of southwest Sri Lanka. The additional primary data was further collected island wide using opportunistic observations and photographic records through the multiple channels of authors’ research networks [13].

### Collection and analysis of data on crimes and rescue efforts related to Indian pangolins

**Fig. 2:**
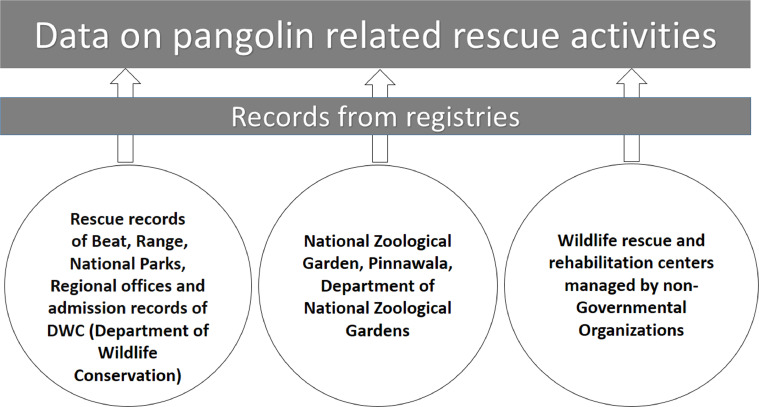
Method used to collect data on pangolin related rescue activities.

The number of crime and rescue records per unit period is a good indicator which shows the level of threat for a species [14]. Wild life rescue and crime records are critically important in the evaluation of conservation status of any species distributed over extensive geographic areas where direct observations are time consuming and costly [15]. Rescue activities are always coupled with the process of rehabilitation except in a case of death of an animal just after the rescue. The records of wildlife rescue centers are critically important in the evaluation of conservation needs. In addition, the admission databases of veterinary hospitals can be used to collect the data on frequency of rescue records. [16]. The errors that could occur due to absence of rescue and crime databases in some of the stakeholder institutions or loopholes in data recording processes can be reduced by collecting the rescue and crime data from multiple data sources [17]. The findings of the temporal and spatial variations of wildlife crime and rescue events are important in the implementation of conservation management programmes. The effectiveness of conservation management activities is directly related to the exact identification of threats and conservation needs [18]. Island-wide crime and rescue records were respectively collected from the offense records and rescue records maintained by the Beat office, Range office, Regional office and the National Park office of the Department of Wildlife Conservation (DWC) Sri Lanka (Figs. 1 and 2). The records on attempted smuggling of pangolin scales and other derivatives were collected from the Department of Customs, Sri Lanka (Fig. 1). The pangolin related crimes and raids handled by the Department of Police under special operations were also collected from the police records of the respective police stations (Fig. 1). The rescue records maintained by the Zoological Garden, Pinnawala,

(7° 18′ 2″ N, 80° 23′ 18″ E), of the Department of the National Zoological Gardens, Sri Lanka were also collected. Further records on the rescue activities were collected from the wildlife healthcare facilities maintained by the DWC and the similar facilities maintained by the nongovernmental organizations (Fig. 2). The rescue and crime records reported by the mass media were also collected after verifying the authenticity from respective authorities (Figs. 1 and 2). Due to the difficulty of retrieving old records, only the cases reported between January 2000 and December 2018 were considered for this study. GPS coordinates of the places where the crimes and rescue activities happened were used to generate maps that show the abundance of crime and rescue activities using the Kernel density tool of Arc GIS 10.1 Software package. Geographical profiling using the spatial data related to the crime and rescue activities are vital for the effective antipoaching interventions and identification of conservation priorities on different landscapes [19]. The graphs of the number of crimes verse year and number of rescue verse year were produced to identify the trend patterns of the pangolin related crimes and rescue activities throughout the study period. In addition, the data from the registries of above institutions were further tabulated for the secondary use.

### Collection and analysis of data on distribution

**Fig. 3:**
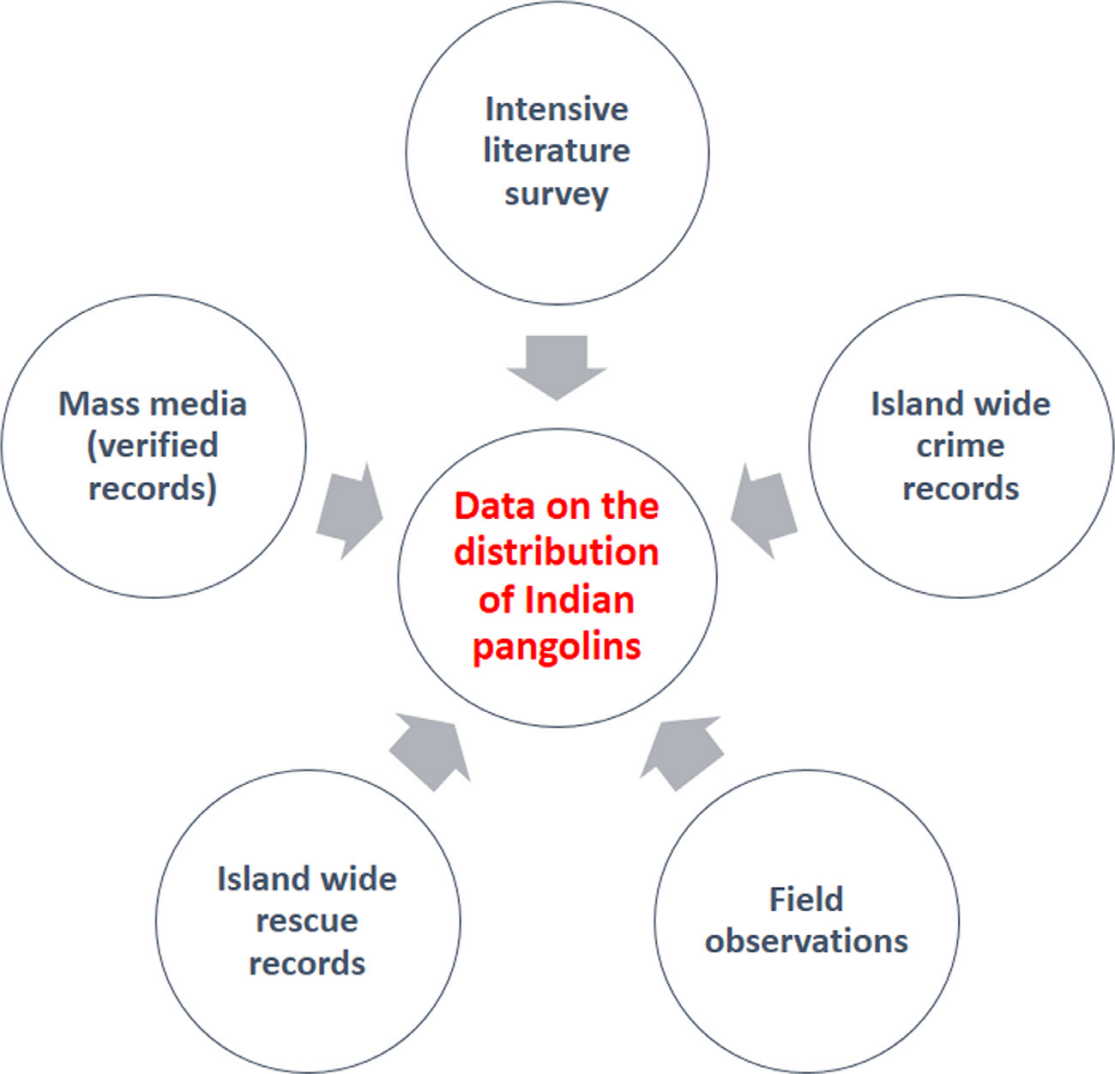
Method used to collect data on distribution of Indian pangolins.

Since the pangolins are distributed over many types of fragmented habitats over the country, direct observations are not feasible. As the species is elusive, the records were few in number and the community awareness on the species is at a poor level [20]. The confirmed field records (record of live or dead specimens, photographs from camera traps etc.) and the records from the crime and rescue data collections were used to record the distribution of the species in island wide. The camera trap surveys were also conducted in some habitats, which presently lack, of evidences for the presence of the species, even though their presence was abundantly recorded in past decades. However, camera trap surveys were limited to certain localities due to the high risk of theft of cameras by poachers and other trespassers to forests. We further developed a web page under our research center at the University of Sri Jayewardenepura website with our research content on pangolins and contact details. Through the above website, we built a field researcher network to share findings related to pangolins and were able to obtain a significant number of records. For each of the above distribution record the location

(Village/Suburban area/City), approximate GPS coordinates, habitat type according to the IUCN classification, altitude (in meters), date of recorded, and source of data (field observation, camera trapping, dead or alive Specimens, DWC/other government or nongovernmental institutional record, record from literature) were recorded. Similar to the mapping of crime and rescue records, the Kernel Density tool of the Arc Gis 10.1 software package was used to produce a map of recorded areas. The distribution records were further analyzed by overlaying points of recorded localities on the mammalian habitats map proposed by Eisenberg and McKay [21] to understand the type of habitats of the Indian pangolins (Fig. 3).

### Semi structured interviews

When considering the number of wildlife related crimes and rescue activities, the number of official records are rather less compared to the actual numbers; therefore use of alternative data sources were critically important in collecting crime and rescue data related to pangolins [22]. The collection of data on elusive nocturnal species is generally a tedious process where direct observation are limited [23]. The studies on the distribution and conservation status of pangolins in the Bangladesh also used semi-structured interviews with different stakeholder groups to collect more data on the nature of pangolin related crimes [24]. At the outset, a pilot study was conducted for a selected small group of individuals by giving them a set of questions initially planned for the interview. Later those questions were further developed to extract more details related to the objectives of the study [1]. Officers of the DWC, villagers, and hunters living around the selected protected areas were interviewed to get a better understanding on pangolin related crimes, niche markets, trafficking pathways, loopholes in law enforcement and several other related aspects. The semi-structured interviews were used as research tool to collect, data from interviewees. The studies by the Cooper et al. [18] has recommended interviewing multiple stakeholder groups such as specialists in wildlife crime management, staff of law enforcement bodies, field naturalists, veterinary personnel and hunters to unveil true facts about of crimes related to any species as wildlife crimes are not isolated events. Since the study was supported by the Department of Wild life Conservation, Sri Lanka and the Research Council of the University of Sri Jayewardenepura, Sri Lanka, no further ethical clearance was needed to carry out the structured interviews especially with local communities. The verbal consent was obtained from respondents before the structured questionnaire was administered, and the respondents were informed that they are free to opt-out of the interview at any point. The individuals below the age of 18 years were not interviewed [1]. When carrying out the interviews with the hunters who killed the pangolins for domestic consumption or selling, their personal involvement was not directly questioned. Instead, they were mainly questioned on the existing exploitation trends related to pangolins [25]. At the bingeing of the interview, a photograph of an Indian Pangolin was shown to each interviewee to verify exact species that they are going to detail throughout the interview. This is to avoid giving inaccurate details lead by misidentification of other species as pangolins [26]. The interviews were conducted using the “Sinhala” language and later the information was translated to the English. Two-person teams that trained on predefined interview framework were deployed to collect the data through interviews. Open-ended questions were asked from the interviewees enable them to express their experiences and knowledge more openly [27,28]. The interviewers were trained to sought additional important information by asking for more clarifications in cases of revealing new knowledge or experiences by interviewees. One to three officers were interviewed from each of the visited DWC offices.

The natural history of the species is also critical in the study of consumption patterns, which lacks published information. Therefore interviewing tribal groups livelihood of hunting was essential for the study [24]. To gain understanding on the traditional uses of the pangolins and their derivatives, an interview was carried out with the chief of the ‘Vedda’; the only forest dwelling indigenous community that has a traditional livelihood based on hunting for subsistence. Since hunting is illegal in protected areas of the country and the nature of sensitive information sought, the snowball sampling method was used to find hunters for the interview process in which the prospective interviewees were approached through the reference and contacts of the previous interviewee [29]. The long run or effectiveness of any conservation programme has a direct relation with the participation and perceptions of the different social groups that closely interact with the targeted species. The findings from ethnozoological surveys have a wider application in planning conservation strategies and harmonizing them with the rituals of social communities associated with the species [30]. The villagers and hunters living in the three major study sites (Yagirala, Wilpattu, and Yala sites) and nearby areas of selected national parks/protected areas (Girithale, Galoya, Angammadilla, Horton Plains, Maduru Oya, Uda Walawe, Minneriya, Kaudulla, Kumana, Hikkaduwa, Lunugamwehera, Kalawewa, Lahugala and Ritigala) were interviewed to gather information using the above interview techniques. A total of 148 individuals; which included 46 DWC officers, 67 villagers, 31 local hunters, and 4 veterinarians/caretakers at animal rescue centers were interviewed for this study.
